# Advanced Access in the Family Health Strategy: progress or setback for the nursing team?

**DOI:** 10.1590/0034-7167-2024-0481

**Published:** 2026-01-26

**Authors:** Isabela Oliveira Almeida, Débora de Souza Santos, Dalvani Marques, Eliete Maria Silva, Paula Cristina Pereira da Costa, Fernanda Mota Rocha, Giovanne Bento Paulino

**Affiliations:** I Universidade Estadual de Campinas. Campinas, São Paulo, Brazil

**Keywords:** Primary Health Care, Health Services Accessibility, Workflow, Nursing, Team, Qualitative Research., Grupo de Enfermería, Atención Primaria de Salud, Estrategias de Salud Nacionales, Accesibilidad a los Servicios de Salud, Flujo de Trabajo.

## Abstract

**Objectives:**

to analyze the implementation of the Advanced Access method in a Family Health Unit from the perspective of the team’s healthcare professionals.

**Methods:**

qualitative research, using participant observation techniques and semi-structured interviews, carried out with 19 members of the multidisciplinary team of a Basic Health Unit in the countryside of São Paulo from February 2019 to March 2020.

**Results:**

the results indicate satisfaction and greater problem-solving, but with compromised longitudinality and comprehensiveness of care due to deficit in team size, population size, and care for chronic patients. The categories of analysis found were “The context of Advanced Access in the access dimensions” and “Nursing as a power for the Advanced Access model”.

**Conclusions:**

the implementation of Advanced Access and its success must consider the history and context of Primary Health Care in each unit and in municipal policy.

## RESUMEN

**Objetivos:**

analizar la implementación del método de Acceso Avanzado en una Unidad de Salud Familiar desde la perspectiva de los profesionales de salud del equipo.

**Métodos:**

investigación cualitativa, mediante técnicas de observación participante y entrevistas semiestructuradas, realizada con 19 miembros del equipo multidisciplinario de una Unidad Básica de Salud del interior de São Paulo, entre febrero de 2019 y marzo de 2020.

**Resultados:**

los resultados indican satisfacción y mayor capacidad de resolución de problemas, pero con una longitudinalidad e integralidad de la atención comprometidas debido a la discrepancia entre el tamaño del equipo, el tamaño de la población y la atención a pacientes crónicos. Las categorías de análisis encontradas fueron “El contexto del Acceso Avanzado en las dimensiones del acceso” y “Enfermería como motor del modelo de Acceso Avanzado”.

**Conclusiones:**

la implementación del Acceso Avanzado y su éxito deben considerar la historia y el contexto de la Atención Primaria de Salud en cada unidad y en la política municipal.

## INTRODUCTION

The prioritization of Primary Health Care (PHC) in Brazil occurred based on the expansion of the Family Health Strategy (FHS) and the approval of the Brazilian National Primary Care Policy, through Ordinance 648 of March 28, 2006, issued by the Ministry of Health and updated by Ordinances 2,715/MO/MoH of November 17, 2011 and 2,436 of 2017. It focuses on the implementation and reaffirmation of the Brazilian Health System (In Portuguese, *Sistema Único de Saúde* - SUS) fundamental principles, such as universality, equity, and comprehensiveness, as well as its organizational principles, such as regionalization and hierarchization, decentralization and single command, and popular participation. Thus, it seeks to guarantee health as a right of every citizen, without exception, considering each individual’s distinct and specific in a singular, comprehensive, and socioculturally contextualized manner^(1-3)^.

The most traditional access method for providing healthcare services, which characterizes PHC as the gateway to the care network, is based on a welcoming approach with qualified listening, which prioritizes emergency cases through risk stratification based on flow and care protocols. After this stratification, urgent patients are directed to the necessary care, and non-urgent requests are directed to same-day scheduling, scheduled for a future date, or referred to another care facility^(4)^. The population’s needs, considering their specificities, must be met by spontaneous demands and scheduled agendas so that reception is characterized as a strategy for expanding access to PHC, as well as the extension of service hours, the incorporation of digital tools and the diversity of forms of access^(4)^.

The Advanced Access (AA) model emerged as a reconstruction of the scheduling system of a saturated PHC in northeastern California in 1990^(4)^, and has been implemented in other health facilities in different countries since then. The AA model is based on the principle of “doing today’s work, today”, seeking to balance patient demand with healthcare provision by their referring provider. This means that incoming patients will have their problem resolved on the same day they seek care, whether their case is urgent, routine, or preventative^(5)^.

Therefore, only prenatal and well-child visits, for instance, are scheduled in advance and held on days with less frequent visits. Appointments should last approximately 15 to 20 minutes to avoid overloading the service and long waiting lists^(6,7)^. According to the AA logic, waiting lines for a scheduled appointment are abolished, as waiting time will be reduced to “today”, which also addresses absenteeism. According to authors studying AA implementation in Brazil, for it to be effective, the service offered must continue to follow the Brazilian National Primary Care Policy and SUS principles. Consultations should focus on comprehensiveness, not limited to complaint-based management^(6)^.

Although scarce, there are already studies in Canada and in southeastern Brazil that show a higher score in the quality of healthcare services that use AA as a model, a factor related to greater agility of care and greater adaptation to users’ needs^(8,9)^. The model’s implementation in Brazil is recent, and has occurred without adequate preparation or awareness among teams due to the lack of national references for the model. There are records of implementation using guidance booklets without adequate in-depth discussion about access and reception^(10,11)^.

Given the relevance of investigating the contributions, limitations, and challenges of AA in the Brazilian PHC context, a study that describes the implementation process in a practical manner is necessary.

## OBJECTIVES

To analyze the implementation of the AA method in a pilot Family Health Unit (FHU) from Family Health team healthcare professionals’ perspective.

## METHODS

### Ethical aspects

Since this is research involving human beings, it was necessary for this study to be assessed by the *Universidade Estadual de Campinas* Research Ethics Committee and the Municipal Health Department of Campinas, in order to confirm compliance with ethical principles, in accordance with Resolution 466 of the Brazilian National Health Council published in 2012^(12)^.

### Theoretical-methodological framework

The research was based on the health access theoretical framework described by Giovanella and Fleury (1996), considering access as a central category for analyzing the interrelationships between users and healthcare services, using specific dimensions that describe the adequacy between users and the healthcare system in the research setting. To better understand the AA model and its functioning, as well as to analyze the model in the context of the studied unit, the framework proposed by Murray and Tantau (2000) was used.

### Study design

This is research originating from a master’s dissertation^(13)^. This is a qualitative study, considering that it involves human relationships, workers’ perspective, care structuring, and patient access to healthcare services. This approach aims for a scientific investigation focused on the subjective nature of the object analyzed, considering all individual experiences and particularities, in which language in various forms of expression becomes the material for analysis^(14)^. The research met the items recommended by the Standards for Reporting Qualitative Research^(15)^.

### Methodological procedures

#### Study setting

The research setting was a pilot FHU in AA implementation, a component of the basic health network of the municipality of Campinas, a city located in the countryside of the state of São Paulo that has approximately 1,200,000 inhabitants and 66 Basic Health Units divided into five districts^(16)^. The Health Center (HC) in question has a population of 9,387 people (2020) in its coverage area, seven neighborhoods and approximately 150 sites. It is a population mostly in the adult and young adult age range (5,603)^(16)^. Its area belonged to another HC until mid-2000, when it was implemented.

Its coverage area has rural characteristics with urban sprawl. There is a significant social contrast within the territory, where vulnerable social conditions coexist with high-end condominiums. Most of the population lacks access to sewage treatment, and there are few bus routes. The current headquarters is rented and adapted, with inadequate infrastructure and insufficient rooms.

#### Data source

All members of the FHU team participated in the study (as of March 2020), totaling 19 participants: the FHU coordinator; two nurses; a family health physician; a pediatrician; a psychologist; four nursing technicians; three nursing assistants; five community health workers; and a pharmacy technician. Of the 19 participants, only one was male. Their ages ranged from 32 to 66 years; ten participants identified as white; eight participants identified as having black/brown skin color; and one participant identified as having yellow skin color. The inclusion criterion was being a member of the unit’s health team.

#### Data collection and organization

Participant observation and semi-structured interviews were used as data collection techniques. The researcher was immersed in the daily life of the studied setting as a master’s student. “Participant observation” allows for interaction between the researcher, the subjects observed, and the context in which they live^(17)^. To record the observations, a field diary was used to take notes on the behavior of the individuals involved, formal and informal conversations, the interlocutors’ statements regarding the points investigated, and the researcher’s personal impressions, which were essential for data collection and analysis^(18)^.

In participant observation, a researcher visited the FHU for 72 days, from February 2019 to March 2020, staying an average of three hours daily. During this time, she recorded staff’s and patients’ behaviors and feelings; observed the waiting room and the flow of patients within the unit; conducted conversations with staff members; and observed meetings. Documents and reports were also analyzed as participant observation components, especially those containing staff information about AA implementation, such as meeting minutes and staff agendas.

Semi-structured interviews aimed to investigate characteristics, perceptions, and feelings about the AA model and the unit’s operations. They were conducted in person at the unit with team members involved in the implementation process. The trigger question used was: what are the main changes observed in the HC after AA implementation? The interviews lasted an average of ten minutes and were recorded and transcribed verbatim by the researcher. To ensure participants’ anonymity, they were identified by the initial of the word “team”, the letter “T”, followed by Arabic numerals, according to the order in which the interviews were conducted.

The collected data is stored through cloud computing in the form of photos, scans, written records, audios, and transcripts.

#### Data analysis

To analyze the empirical material, the hermeneutic-dialectical method, applied in health studies, designed by Minayo, was used^(19)^. Hermeneutics and dialectics constitute “two paths through which the current debate on the question of method develops in a sphere that transcends the fragmentation of scientific procedures in general”^(20)^.

First, field data were organized by mapping recording transcription, rereading the material, and organizing reports and participant observation data. After rereading the material, the data were classified to identify what was relevant and, from there, develop specific categories. The first category, “The context of Advanced Access in the access dimensions”, was developed by relating the challenges and potential of AA to the access dimensions addressed by Giovanella and Fleury^(19)^. The second category, “Nursing as a power for the Advanced Access model”, was developed based on collected data that identified nursing as a strategic factor in the success of the implemented model. In the final analysis, connections were made between the collected data and the theoretical frameworks.

## RESULTS

### The context of Advanced Access in the access dimensions

Due to the population’s characteristics, spontaneous demand has always been high. However, although the team constantly adapted its schedule to improve care provision, it prioritized the programmatic schedule over spontaneous demands. However, most patient complaints were acute, often work-related, reflecting the fact that the population is 50% young adults.

After realizing that the unit’s schedule did not meet the population’s needs, the team began discussing new scheduling alternatives to improve care and flow at the unit.

In March 2018, a resident returned to the unit after completing an internship in Florianópolis, Santa Catarina, where he had experience with AA at a unit very similar to the one he was studying. After studying the facility’s work and schedule, articles, and documents related to AA, the resident brought the suggestion to a staff meeting. It was a scheduling method that fit the unit’s needs, relieving the burden on the reception team and physicians, and directly involving nurses in care delivery.

The team spent several months studying, discussing, and adapting the AA proposal. The transition period was difficult and laborious, due to the need to balance the existing schedule with the spontaneous and free demand.

*They say it was a very stressful, very difficult period* […] *when we read AA literature, this transition period is always difficult and laborious, but it is necessary to clear the agenda and start AA.* (T3)

There were many discussions in team meetings about AA, its flow and operation, as well as the challenges and difficulties the team encountered during the process. After AA was implemented in 2018, the team reports that more and more people are seeking the unit. Spontaneous demand has increased, a consequence of the loss of health insurance plans and the migration of families to the region in search of opportunities. Inversely proportional to the growth in demand is the shortage in the number of professionals on the health team, which has fallen significantly. The HC had only one team.

*Right now, I can say HC is experiencing the worst crisis we’ve ever experienced in terms of staff. A very small team right now* […]. *We work from the perspective of a Family Health Program, but we don’t do a Family Health Program because we have a team to assist 9,000 people.* (T6)

The schedule operates with 60% of appointments open to spontaneous demand and 40% scheduled appointments. The unit’s sole physician’s schedule is a maximum of two prospective months, but due to patient volume, she often has to take a slot reserved for spontaneous demand for a scheduled patient.

Another factor contributing to the high demand for the unit is its distance from other healthcare services, as the unit is located 14 km from the nearest hospital and 23 km from the main emergency room. The population’s socioeconomic status and the scarcity of public transportation in the region make access to other services difficult.

The unit’s Local Health Council has shown itself willing to question neglect and delays, and demand improvements to the service, and is aware that it must organize and fight for its rights.

Staff members’ opinions on the AA model are diverse and divided. Some consider AA to be an appropriate model for the HC population and report that patients leave the unit satisfied with having their issues resolved on the same day.

*For the HC population, who seek out and greatly need the service, it’s the only service in the region, so people turn to it for everything. So, at this point, with this population, I don’t see how a closed schedule could be better.* (T3)

Others, however, argue that AA is not the right model and that patients dislike the wait. Observations revealed the length of time patients spend in the waiting room awaiting appointments.

*Patients complain a lot. They don’t like it, they like making appointments, coming* […] *they have to stay here all morning. Sometimes they waste the whole morning to be seen at noon, an hour in the afternoon, sometimes even until they come back, so they don’t like it. And neither do we.* (T8)

Different workers’ perceptions coincide on the main problem of AA for the team: the lack of HR, which causes an imbalance in the supply/demand relationship.

*Advanced Access is very good for patients. It would be great for us too, but if we had a more complete team, because it removes barriers to care. However, for it to be seamless, we would need HR, and we don’t.* (T1)

Among the negative aspects highlighted was the workload, resulting from an imbalance in demand and supply. Resolution was also a key point of discussion, with differing opinions. The constantly crowded waiting room and frequent return patients were among the points of contention.

For some team members, the limited time available to care for that individual is limited to a complaint-and-management consultation, addressing only patients’ acute problem. This leads to a reflection frequently observed in the observations and interviews: what used to be a HC now feels more like an Emergency Room (ER).

*The demand is so high, it will become an ER, access will not be advanced enough to look at the whole, it will only look at that little thing* […]. (T7)

Other officials, however, disagree with this view.

*The team’s relationship with AA needs to improve. People think the AA will turn the HC into an ER, and it isn’t. We know the person we’re seeing. They’ll always come back to me; I’m their primary care physician; they’ll never be discharged.* (T3)

Another common concern is the care of patients with chronic diseases. In primary care, the idea is for patients to seek care whenever they want, so care for these patients may not be comprehensive, long-term, or follow-up, which undermines the long-term nature of care, an essential attribute from a PHC perspective.

*I think patients are becoming too scattered, especially chronic patients, diabetics, hypertensive patients. I think it’s getting loose, I don’t know. It’s good that it provides resolution at that moment. The patient leaves feeling happy, but they left, I don’t know* […] *and what about their return?* (Nursing technician, 38 years old)

Given these perceptions, the team itself conducted a survey of 700 patient records at the hospital during weekly meetings to characterize the population, identifying approximately 200 patients with hypertension and diabetes. After an assessment, the team found that 80% of these chronic patients had seen a physician within the 12-month period, a rate considered positive for the hospital’s reality.

### Nursing as a power for the Advanced Access model

With AA implementation, the work process has not changed significantly for nursing technicians and assistants. The main difference is the elimination of appointment scheduling, which previously was done by them once a month.

At AA, technicians and assistants triage patients who arrive at the unit and are seen on the same day, referring them for medical or nursing consultations, or providing specific guidance. Along with community health workers, these are the team members who have the most direct contact with patients, frequently listening to their opinions on the model.

With AA implementation and the overload of the unit’s few physicians, the work process of the unit’s sole nurse was also discussed in team meetings and interviews. It was suggested that the nurse participate in the screening performed by technicians, which would enrich the process and speed up patient care flow.

*Nurses have the technical capacity to assess, listen to the issue, and resolve many issues. Now, nursing technicians don’t. Of course, we provide qualified listening, but we can’t resolve the issue alone. A nurse would need to be present; qualified listening would need to be done with nurses.* (T2)

Thus, nursing work’s potential and ability to competently perform various functions that would benefit AA in the unit also came into focus. However, a high patient demand would also end up overwhelming nurses, and the population’s culture also influenced their work process.

*Most people don’t like* [the AA] *because they come hoping to see a physician, and then the physician’s openings run out, so the patient ends up seeing a nurse, and they don’t like it. They don’t like it, they want to see a physician, because they have that culture.* (T7)

## DISCUSSION

### The context of Advanced Access in the access dimensions

Access to health is defined as the timely use of services in order to meet users’ needs^(21)^. In order to support the understanding of access as a way of approaching healthcare services and matching users’ and care system’ needs, specific dimensions that are interrelated were described, with access as the central category of analysis: economic; technical; political; and symbolic^(21,22)^.

According to Giovanella and Fleury, the relationship between supply and demand is one of the access dimensions analysis, called the economic dimension, which has as its main basis the relationship between healthcare services that are offered and the population’s health needs, called the supply/demand relationship, which must be balanced through the creation of government policies^(21,23)^. According to FHS, the Basic Health Unit (BHU) must be located in the territory in which it operates, with a population of up to 12,000 inhabitants. Each multidisciplinary team must be responsible for a maximum of 3,000 inhabitants so that the supply/demand ratio is balanced^(24)^. Taking this framework into account, the supply/demand ratio of the studied HC is markedly disproportionate. Two more complete Family Health teams would be needed to cover the population size of the assigned territory.

A study conducted with 127 clinics in Canada that use AA as an access model shows that, among the main strategies adopted to balance supply and demand are identifying demand patterns, providing additional consultations to clear backlogs and varying consultation modalities (mainly by telephone)^(25)^.

As professionals point out, the lack of human resources contributes to the imbalance between limited supply, high demand, and fragmented care, hindering comprehensive care provision and guarantee. Comprehensive care, as enshrined in the SUS, ensures that consultations focus on the individual and are not based on a complaint-based approach^(2)^. Daily practices of the supply/demand relationship must therefore be based on the comprehensiveness paradigm^(22)^.

Regionalization is also a factor that aims to provide more effective healthcare in units with different care profiles organized hierarchically^(26)^. This organization, established in a health microregion, guarantees the population a variety of actions and services that could not be offered otherwise. This is addressed in the technical dimension of access analysis^(21,24)^, which encompasses the planning and organization of a healthcare service network. This dimension is compromised in the context of the HC due to its distance from other healthcare services and the difficulties the population faces in accessing the facility.

According to the political dimension of access, community participation is extremely important. Based on the logic of health awareness, health must be achieved through individual and collective action, as it is a citizen’s right. Furthermore, building access must happen through citizen participation and social control. A well-positioned and active local health council can have a positive influence on access to the unit. Action must also be based on the population’s understanding of the social determinants of the health-disease process and the factors that define the social organization of health practices^(22,24)^. During team meetings, the importance of the local council’s participation in implementing AA was emphasized, with members expressing their views on its positive and negative aspects and the necessary changes to improve public access to the new model.

The contradictions present in the health team’s statements regarding AA are in accordance with the symbolic access dimension, in which social subjects, collectively determined by their representations, are formed by historical subjects, who are initially collective subjects^(21)^. Each subject, in its collective dimension, has a material and spiritual reality that is related to the production of ideas, representations and consciousness^(24,26)^. This dimension also highlights the importance of the team becoming familiar with AA and its theoretical framework, since a lack of knowledge can generate doubts and influence the model implementation and functioning^(6)^. When implementing the model in Canadian units, strategies used were folders, practical courses and simulations, in addition to group discussions and the provision of books and references^(25)^. Some of the statements and issues raised in the interviews could be resolved with a more in-depth theoretical approach. A study conducted during AA implementation in a primary care unit in the city of São Paulo highlighted the team’s lack of knowledge about the model as a hindrance to its implementation. Interviewees reported difficulty implementing some of the innovations proposed by the team due to a lack of understanding of the model^(27)^.

### Nursing as a power for the Advanced Access model

The nursing team’s role in PHC is broad and diverse, encompassing clinical, educational and managerial practice actions in the unit^(2)^. Teamwork, responsiveness in care, connection with patients, and affinity for work are all factors that contribute to universal access to healthcare, one of the goals of FHS. However, increased nursing workloads pose a challenge to the effectiveness of this access, compounded by work overload, excess demand, structural issues in units, and gaps in the care network^(28)^. The nursing work process, aiming at this universality of access and also at care centered on the population’s health needs, needs to be focused on users, not on individual procedures^(28)^.

AA implementation has allowed patients access to the multidisciplinary schedule, which values and reaffirms the role of these professionals in PHC, as well as a break with the biomedical model. A 2020 study conducted at a primary care unit in the municipality of Diadema, São Paulo, highlights nurses’ autonomy as a key factor in the effectiveness of AA in the unit. In this case, physicians’ schedules were overcrowded, and some cases were redirected to nurses’ schedule, which increased case resolution and reduced the waiting list for care^(7)^.

The literature has demonstrated nursing’s potential for developing AA, with increased autonomy, problem-solving ability, and engagement with users’ needs, improving access to PHC. A study that assessed the problem-solving ability of nursing consultations with AA showed that nurses use technical and scientific knowledge to meet the diverse needs of the population, providing solutions in 87.7% of consultations, achieving a higher resolution rate than the Ministry of Health’s recommended 85%^(29)^. In the observed scenario, the unit nurse did not have an agenda favorable to resolving demands, and changing this factor was highlighted by the team as essential for the change to AA.

### Study limitations

Tension among the team due to overload and divergent opinions may have affected the data collected, and the onset of the COVID-19 pandemic interrupted the researcher’s visits to the site to collect data.

### Contributions to nursing, health or public policy

The results of this study can help us understand the model in a practical manner, highlighting its challenges. Furthermore, it also contributes to a better understanding of nursing’s potential for action within the context of access to health and care services, as well as service organization and workflow.

## FINAL CONSIDERATIONS

According to existing literature, the AA model faces many challenges in the context of the HC studied. Although appropriate for the characteristics of the unit’s population, the imbalance between supply and demand becomes an obstacle to adequate and comprehensive care.

There is tension within the team due to the disproportion between the team size and the number of users and services, resulting in significant overload. Furthermore, the team has significant differences of opinion regarding the model, with opposing views.

Patients appreciate having their problems resolved on the same day, but not everyone can have an appointment due to limited availability. Concerns about continuity of care and assistance for chronic patients were also a key point of discussion, as was the fear that the service would resemble an ER.

Nursing appears as a possible enhancer of the model, when appropriated of its resolution capacity and technical-scientific knowledge that meets users’ needs.

The AA model stands out as a possibility for organizing agendas, but its implementation and success must consider the history and context of PHC in each unit and in municipal policy, which must be aligned with the SUS and Family Health principles as the organizer of the model.

## Data Availability

The research data are available only upon request.
