# Supplementary material for: Advanced Access in the Family Health Strategy: progress or setback for the nursing team?
Source: Rev Bras Enferm. 2026 Jan 26;79:e20240481. doi: 10.1590/0034-7167-2024-0481 (PMC12844541; doi:10.1590/0034-7167-2024-0481)
Supplement: Translation

# Translation: Acesso Avançado na Estratégia Saúde da Família: avanço ou retrocesso para a equipe de enfermagem?

**DOI:** 10.1590/0034-7167-2024-0481pt

**Authors:** Isabela Oliveira Almeida, Débora de Souza Santos, Dalvani Marques, Eliete Maria Silva, Paula Cristina Pereira da Costa, Fernanda Mota Rocha, Giovanne Bento Paulino

**Affiliations:** I Universidade Estadual de Campinas. Campinas, São Paulo, Brasil

**Keywords:** Equipe de Enfermagem, Atenção Primária à, Saúde, Estratégia Saúde da Família, Acesso aos Serviços de Saúde, Fluxo de Trabalho.

## Abstract

**Objetivos:**

analisar a implantação do método Acesso Avançado em Unidade de Saúde da Família na perspectiva dos profissionais de saúde da equipe.

**Métodos:**

pesquisa qualitativa, com utilização das técnicas de observação participante e entrevistas semiestruturadas, realizada com 19 membros da equipe multiprofissional de Unidade Básica de Saúde do interior de São Paulo de fevereiro de 2019 a março de 2020.

**Resultados:**

os resultados apontam para satisfação e maior resolutividade dos problemas, porém com comprometimento da longitudinalidade e integralidade do cuidado, devido ao déficit entre o tamanho da equipe, de sua população e cuidados com pacientes crônicos. As categorias de análise encontradas foram “O contexto do Acesso Avançado nas dimensões do acesso” e “A enfermagem como potência para o modelo de Acesso Avançado”.

**Conclusões:**

a implantação do Acesso Avançado e seu sucesso devem considerar a história e o contexto da Atenção Primária à Saúde em cada unidade e na política municipal.

## INTRODUÇÃO

A priorização da Atenção Primária à Saúde (APS) no Brasil ocorre a partir da expansão da Estratégia Saúde da Família (ESF) e da aprovação da Política Nacional de Atenção Básica, por meio da Portaria nº 648, de 28 de março de 2006, emitida pelo Ministério da Saúde e atualizada por meio das Portarias nº 2.715/GM/MS, de 17 de novembro de 2011, e nº 2.436, de 2017. Tem como foco a implementação e reafirmação dos princípios fundamentais do Sistema Único de Saúde (SUS), como universalidade, equidade e integralidade, assim como seus princípios organizacionais, como regionalização e hierarquização, descentralização e comando único, e participação popular. Assim, busca garantir a saúde como um direito de todo cidadão, sem exceções, considerando as necessidades distintas e específicas de cada ser, de modo singular, integral e contextualizado socioculturalmente^(1-3)^.

O método de acesso mais tradicionalmente usado para se oferecer os serviços em saúde e que caracteriza a APS como porta de entrada à rede de atenção se baseia no acolhimento com escuta qualificada, que visa atender prioritariamente casos de urgência/emergência por meio de classificação de risco baseada em fluxo e protocolos de atendimento. Após essa classificação, o paciente urgente é direcionado para o atendimento necessário, e demandas não urgentes são direcionadas para consultas no mesmo dia, agendadas para um momento futuro ou encaminhadas para outro ponto de atenção^(4)^. As necessidades da população, considerando suas especificidades, devem ser contempladas pelas demandas espontâneas e agenda programada, de modo que o acolhimento se caracteriza como uma estratégia de ampliação do acesso à APS, assim como a extensão do horário de atendimento, a incorporação de ferramentas digitais e a diversidade de formas de acesso^(4)^.

O modelo de Acesso Avançado (AA) surgiu como uma reconstrução do sistema de agendamento de uma APS saturada no nordeste da Califórnia em 1990^(4)^, e passou a ser implementado em outras unidades de saúde em diferentes países desde então. O AA tem como princípio “fazer o trabalho de hoje, hoje”, buscando equilibrar a demanda dos pacientes com a oferta de cuidados em saúde por seu profissional de referência. Isso significa que o paciente que chega terá seu problema resolvido no mesmo dia em que procurar o atendimento, seja seu caso urgente, rotineiro ou preventivo^(5)^.

Assim, apenas consultas pré-natais e puericulturas, por exemplo, são agendadas previamente e realizadas em dias menos ocupados por demandas espontâneas. As consultas devem durar em torno de 15 a 20 minutos, para evitar sobrecarga do serviço e fila de espera longa^(6,7)^. Segundo a lógica do AA, as filas de espera para uma consulta agendada são abolidas, já que o tempo de espera se reduzirá a “hoje”, o que também resolve o absenteísmo. Segundo autores que estudam a aplicação do AA no Brasil, para que este seja efetivo, o serviço oferecido deve continuar seguindo os princípios da Política Nacional da Atenção Básica e do SUS em suas ações. As consultas devem focar na integralidade, não se restringindo à queixa-conduta^(6)^.

Apesar de escassos, já existem estudos no Canadá e na região Sudeste do Brasil que evidenciam um maior escore na qualidade dos serviços de saúde que usam o AA como modelo, fator relacionado à maior agilidade dos atendimentos e maior adequação às necessidades dos usuários^(8,9)^. A implantação do modelo no Brasil é recente, e tem ocorrido sem o devido preparo e conscientização das equipes diante da falta de referências nacionais sobre o modelo. Existem registros de implantação com o uso de cartilhas de orientação sem a devida profundidade de discussão acerca de acesso e acolhimento^(10,11)^.

Diante da relevância em investigar sobre as contribuições, limitações e desafios do AA no contexto brasileiro da APS, faz-se necessário estudo que descreva o processo de implantação de forma prática.

## OBJETIVOS

Analisar a implantação do método de AA em Unidade de Saúde da Família piloto na perspectiva dos profissionais de saúde da equipe de Saúde da Família.

## MÉTODOS

### Aspectos éticos

Por se tratar de pesquisa que envolve seres humanos, fez-se necessária a apreciação deste estudo pelo Comitê de Ética em Pesquisa da Universidade Estadual de Campinas e pela Secretaria Municipal de Saúde de Campinas, a fim de confirmar o respeito aos princípios éticos, conforme a Resolução nº 466 do Conselho Nacional de Saúde publicada em 2012^(12)^.

### Referencial teórico-metodológico

A pesquisa foi fundamentada no referencial teórico de acesso à saúde descrito por Giovanella e Fleury (1996), considerando o acesso como categoria central para análise das inter-relações entre usuários e serviços de saúde, utilizando dimensões específicas que descrevem a adequação entre os usuários e o sistema de cuidados em saúde no cenário da pesquisa. Como forma de maior entendimento acerca do modelo de AA e seu funcionamento, bem como a análise do modelo no contexto da unidade estudada, foi utilizado o referencial proposto por Murray e Tantau (2000).

### Tipo de estudo

Trata-se de pesquisa oriunda de dissertação de mestrado^(13)^. Estudo de abordagem qualitativa, considerando que implica relações humanas, olhar do trabalhador, estruturação do cuidado e acesso do paciente ao serviço de saúde. Essa abordagem visa uma investigação científica focada no caráter subjetivo do objeto analisado, considerando todas as experiências individuais e particularidades, em que a linguagem em várias formas de expressão se torna material de análise^(14)^. A pesquisa atendeu aos itens recomendados pelo *Standards for Reporting Qualitative Research* ^(15)^.

### Procedimentos metodológicos

#### Cenário do estudo

O cenário da pesquisa foi uma Unidade de Saúde da Família piloto na implantação do AA, componente da rede básica de saúde do município de Campinas, cidade localizada no interior do estado de São Paulo que possui cerca de 1.200.000 habitantes e 66 Unidades Básicas de Saúde divididas em cinco distritos^(16)^. O Centro de Saúde (CS) em questão possui uma população de 9.387 pessoas (2020) em sua área de abrangência, sete bairros e aproximadamente 150 sítios. É uma população majoritariamente na faixa etária de adulto e adulto jovem (5.603)^(16)^. Sua área era pertencente a outro CS até meados do ano 2000, quando foi implantado.

Sua área de cobertura possui características rurais com manchas urbanas. Existe um contraste social grande no território, em que condições sociais vulneráveis coexistem com condomínios de alto padrão. A maioria da população não tem acesso a tratamento de esgoto, e há poucas linhas de ônibus. A sede atual é alugada, adaptada, com estrutura inadequada e salas insuficientes.

#### Fonte de dados

Participaram da pesquisa todos os membros da equipe da Unidade de Saúde da Família (até março de 2020), totalizando 19 participantes: a coordenadora da Unidade de Saúde da Família; duas enfermeiras; uma médica de Saúde da Família; uma pediatra; uma psicóloga; quatro técnicos de enfermagem; três auxiliares de enfermagem; cinco agentes comunitárias de saúde; e uma técnica de farmácia. Dos 19 participantes, apenas um era do gênero masculino. As idades variaram de 32 a 66 anos; dez participantes se identificaram como brancos; oito participantes se identificaram como como pretos/pardos; e um participante se identificou como amarelo. Foi critério de inclusão ser membro da equipe de saúde da unidade.

#### Coleta e organização dos dados

Como técnicas para coleta de dados, foram utilizadas observação participante e entrevista semiestruturada. A pesquisadora esteve inserida no dia-a-dia do cenário estudado como aluna de mestrado. A “observação participante” oportuniza interatividade entre o pesquisador, os sujeitos observados e o contexto no qual eles vivem^(17)^. Para registro das observações, foi utilizado diário de campo para anotações sobre comportamentos dos indivíduos envolvidos, conversas formais e informais, manifestações dos interlocutores quanto aos pontos investigados, e impressões pessoais da pesquisadora, sendo essencial para a coleta e análise de dados^(18)^.

Na observação participante, uma pesquisadora frequentou a Unidade de Saúde da Família durante 72 dias, de fevereiro de 2019 a março de 2020, com permanência de (em média) três horas diárias. Nesse tempo, captou comportamentos e sentimentos da equipe e dos usuários; observou a sala de espera e o fluxo dos usuários dentro da unidade; realizou conversas com membros da equipe; e acompanhou reuniões. Documentos e relatórios também foram analisados como componentes da observação participante, especialmente aqueles que continham informações da equipe sobre a implantação do AA, como atas de reuniões e agendas dos profissionais.

As entrevistas semiestruturadas tiveram como finalidade investigar características, percepções e sentimentos sobre o modelo AA, e o funcionamento da unidade. Foram realizadas presencialmente na unidade com profissionais da equipe envolvidos no processo de implantação. A questão disparadora utilizada foi: quais as principais mudanças observadas no CS após a implantação do AA? As entrevistas tiveram duração média de dez minutos, e foram gravadas e transcritas na íntegra pela pesquisadora. Para garantir o anonimato dos participantes, eles foram identificados com a inicial da palavra equipe, a letra “E”, seguida de números arábicos, de acordo com a ordem que se realizavam as entrevistas.

Os dados coletados estão armazenados por meio de computação em nuvem em forma de fotos, digitalização, registros escritos, áudios e transcrições.

#### Análise dos dados

Para análise do material empírico, utilizou-se o método hermenêutico-dialético, aplicado em estudos da saúde, desenhado por Minayo^(19)^. A hermenêutica e a dialética constituem “dois caminhos através dos quais o debate atual sobre a questão do método se desenvolve numa esfera que transcende a fragmentação dos procedimentos científicos em geral”^(20)^.

Primeiro, foi realizada a ordenação dos dados obtidos em campo, mapeando a transcrição de gravações, releitura do material, e organização dos relatos e dos dados da observação participante. Após a releitura do material, os dados foram classificados para que fosse possível identificar o relevante e, a partir disso, elaborar categorias específicas. A primeira categoria, “O contexto do Acesso Avançado nas dimensões do acesso”, foi elaborada relacionando os desafios e potencialidades do AA às dimensões de acesso abordadas por Giovanella e Fleury^(19)^. A segunda categoria, “A enfermagem como potência para o modelo de Acesso Avançado”, foi elaborada através de dados coletados que traziam a enfermagem como estratégica para o sucesso do modelo implantado. Na análise final, foram realizadas articulações entre os dados coletados e os referenciais teóricos.

## RESULTADOS

### O contexto do Acesso Avançado nas dimensões do acesso

Por conta das características da população, a demanda espontânea sempre foi grande. Entretanto, apesar de a equipe sempre adaptar a agenda para melhorar a oferta de cuidado, priorizava a agenda programática às demandas espontâneas. Mas a maioria das queixas dos pacientes eram agudas, muitas vezes relacionadas ao trabalho, o que refletia a população ser 50% de adultos jovens.

Após a percepção de que a agenda da unidade não respondia às necessidades da população, a equipe passou a discutir novas alternativas de agendamento, com a intenção de melhorar o cuidado e o fluxo da unidade.

Em março de 2018, um residente voltou à unidade após realizar um estágio externo na cidade de Florianópolis, em Santa Catarina, onde teve contato com o AA em uma unidade muito semelhante ao serviço estudado. Ao estudar o trabalho e agenda do local, artigos e documentos em relação ao AA, o residente levou a sugestão em reunião de equipe. Era um método de agendamento que se encaixava com o perfil da unidade, desafogando a equipe de acolhimento e os médicos, além de envolver a enfermeira diretamente na assistência.

A equipe passou alguns meses estudando, discutindo e adaptando a proposta de AA. O período de transição foi difícil e trabalhoso, pela necessidade de conciliar a agenda, que já existia com a demanda espontânea livre.

*Dizem que foi um período muito estressante, muito difícil* […] *quando a gente lê a literatura de AA, esse período de transição é sempre difícil e trabalhoso, mas é necessário para zerar a agenda e começar o AA.* (E3)

Foram muitas discussões em reuniões de equipe sobre o AA, seu fluxo e funcionamento, bem como desafios e dificuldades que a equipe apresentou no processo. Após a implantação do AA no ano de 2018, a equipe relata que, cada vez mais, mais pessoas procuram a unidade. A demanda espontânea tem aumentado, consequência da perda de planos de saúde e migração de famílias para a região em busca de oportunidades. De forma inversamente proporcional ao crescimento da demanda, está o déficit do número de profissionais da equipe de saúde, que caiu significativamente. O CS contava com apenas uma equipe.

*Nesse momento, eu posso dizer: o CS vive a pior crise que a gente já passou com relação à equipe. Uma equipe nesse momento muito reduzida* […]. *A gente trabalha numa ótica de Programa de Saúde da Família, mas a gente não faz Programa Saúde da Família porque a gente tem uma equipe pra atender 9 mil pessoas.* (E6)

A agenda funciona com 60% de vagas abertas para demanda espontânea e 40% de agendamentos programáticos. A agenda da única médica da unidade é de, no máximo, dois meses prospectivos, mas, devido à quantidade de pacientes, muitas vezes ela precisa pegar um horário destinado à demanda espontânea para um paciente agendado.

Outro fator que contribui para a grande procura da unidade é a distância de outros serviços de saúde, já que a unidade está localizada a 14 km do hospital mais próximo e a 23 km do Pronto Socorro de referência. A condição socioeconômica da população e a escassez de transportes coletivos disponíveis na região tornam difíceis os acessos a outros serviços.

O Conselho Local de Saúde da unidade se mostrou disposto a questionar descasos e atrasos, e cobrar melhorias para o serviço, e tem consciência de que deve se organizar e lutar por seus direitos.

A opinião dos membros da equipe sobre o modelo de AA é diversa e dividida. Alguns consideram o AA um modelo adequado para o contexto da população do CS, e relatam que os pacientes saem satisfeitos da unidade por terem seus problemas resolvidos no mesmo dia.

*Para a população do CS, que procura muito e precisa muito do serviço, é o único serviço da região, então a população procura ele pra tudo. Então, nesse momento, com essa população que a gente tem, não vejo como uma agenda fechada poderia ser melhor.* (E3)

Outros, porém, alegam que o AA não é o modelo adequado e que os pacientes não gostam da demora. Nas observações, foi possível notar o grande tempo que os pacientes ficam na sala de espera aguardando as consultas.

*Os pacientes reclamam muito. Eles não gostam, eles gostam de marcar consulta, de vir* […] *tem que ficar aqui a manhã toda. Às vezes, perde a manhã toda pra ser atendido meio dia, uma hora da tarde, às vezes até voltar, então eles não gostam. E a gente também não.* (E8)

As percepções dos diferentes trabalhadores são coincidentes no principal problema do AA para a equipe: a falta de RH, que causa o desequilíbrio na relação oferta/demanda.

*O Acesso Avançado, pro paciente, é muito bom. Pra nós, também seria, mas se a gente tivesse uma equipe mais completa, porque com ele você tira a barreira de atendimento, só que, pra que ele ocorresse redondinho, teria que ter RH, e a gente não tem.* (E1)

Entre os pontos negativos apontados, encontra-se a sobrecarga de trabalho, vinda da demanda em desequilíbrio com a oferta. A resolutividade também foi um ponto importante de discussão, com divergências de opiniões. A sala de espera constantemente lotada e pacientes que retornam com frequência são pontos dessa discussão.

Para alguns membros da equipe, o pequeno tempo disponível para atendimento daquela pessoa fica restrito a uma consulta estilo queixa-conduta, resolvendo apenas o problema agudo do paciente. Isso leva a uma reflexão muito presente na observação e nas entrevistas realizadas: o que era um CS, agora parece mais um Pronto-Atendimento (PA).

*A demanda tá muito grande, aí vai virar um PA, o acesso não vai ficar avançado de olhar o todo, só olha aquele negocinho mesmo* […]. (E7)

Outros funcionários, no entanto, discordam desse ponto de vista.

*A relação da equipe com o AA precisa melhorar. As pessoas acham que o AA vai transformar o CS em um PA, e não é. Aquela pessoa que a gente tá vendo, a gente conhece. Ele sempre vai voltar pra mim, eu sou o médico de referência dele, ele nunca vai ter alta.* (E3)

Outra preocupação frequente é o cuidado de pacientes com doenças crônicas. No AA, a ideia é que o paciente busque atendimento quando quiser, de modo que o cuidado com esses pacientes pode não estar sendo integral e com longa duração e acompanhamento, o que descaracteriza a longitudinalidade do cuidado, atributo essencial na perspectiva da APS.

*Eu acho que tá ficando muito disperso os pacientes, principalmente crônico, os diabéticos, hipertensos, acho que fica solto, não sei. É bom que dá resolutividade naquele momento. O paciente já sai até feliz, mas foi embora, não sei* […] *e o retorno dele?* (Técnica de enfermagem, 38 anos)

Diante dessas percepções, a própria equipe realizou, durante as reuniões semanais, um levantamento de 700 prontuários de pacientes do CS, com o objetivo de caracterização da população, sendo estacados aproximadamente 200 hipertensos e diabéticos. Após avaliação realizada pela equipe, foi identificado que 80% desses pacientes crônicos haviam passado em consulta médica dentro do período de 12 meses, taxa considerada positiva para a realidade do CS.

### A enfermagem como potência para o modelo de Acesso Avançado

Com a implantação do AA, o processo de trabalho não se alterou significativamente quando se trata da equipe de técnicos e auxiliares de enfermagem. A principal diferença é a abolição dos agendamentos para consultas, que anteriormente eram realizados pelos mesmos uma vez ao mês.

No AA, os técnicos e auxiliares realizam a triagem dos pacientes que chegam na unidade e são atendidos no mesmo dia, encaminhando-os para consulta médica, de enfermagem, ou realizando alguma orientação específica. Junto com os agentes comunitários de saúde, são os membros da equipe que mais entram em contato direto com os pacientes, ouvindo frequentemente a opinião deles sobre o modelo.

Com a implantação do modelo e sobrecarga dos poucos médicos do serviço, o processo de trabalho da única enfermeira da unidade também foi discutido em reuniões de equipe e nas entrevistas. Foi sugerido que a enfermeira participasse da triagem realizada pelos técnicos, o que o tornaria mais rico e agilizaria o fluxo de atendimento dos pacientes.

*O enfermeiro tem capacidade técnica pra avaliar, ouvir a questão, pra resolver muita coisa. Agora, o técnico de enfermagem não, lógico que a gente faz uma escuta qualificada, mas sozinho a gente não consegue resolver. Teria que ter um enfermeiro junto, teria que ser uma escuta qualificada com o enfermeiro.* (E2)

Assim, também entraram em pauta o potencial do trabalho da enfermagem e sua capacidade de realizar com competência diversas funções que beneficiariam o AA na unidade. Porém, a grande demanda de pacientes também acabaria sobrecarregando a enfermeira, além da cultura da população também influenciar seu processo de trabalho.

*A maioria não gosta* [do AA], *porque eles vêm na esperança de passar com o médico, e aí acabam as vagas de médico, aí o paciente vai passar com a enfermeira, e eles não gostam. Não gostam, eles querem passar com o médico, porque eles têm essa cultura.* (E7)

## DISCUSSÃO

### O contexto do Acesso Avançado nas dimensões do acesso

O acesso à saúde se define como o uso oportuno dos serviços de forma a atender às necessidades dos usuários^(21)^. A fim de apoiar a compreensão do acesso como forma de aproximação com os serviços de saúde e adequação entre as necessidades dos usuários e o sistema de cuidados, foram descritas dimensões específicas que se interrelacionam, possuindo o acesso como categoria central de análise: econômica; técnica; política; e simbólica^(21,22)^.

De acordo com Giovanella e Fleury, a relação entre oferta e demanda é uma das dimensões de análise do acesso, chamada dimensão econômica, que tem como principal base a relação entre os serviços de saúde que são oferecidos e as necessidades de saúde da população, chamada relação oferta/demanda, que deve ter seu equilíbrio possibilitado por meio da criação de políticas de governo^(21,23)^. Segundo a ESF, a Unidade Básica de Saúde (UBS) deve localizar-se no território em que atua com população de até 12 mil habitantes. Cada equipe multiprofissional deve se responsabilizar por no máximo três mil habitantes, de modo que a relação oferta/demanda seja equilibrada^(24)^. Levando em consideração esse referencial, a relação oferta/demanda do CS estudado é acentuadamente desproporcional. Seriam necessárias mais duas equipes de Saúde da Família completas para contemplar o tamanho da população no território adscrito.

Estudo realizado com 127 clínicas do Canadá que utilizam o AA como modelo de acesso mostra que, entre as principais estratégias adotadas para o equilíbrio da oferta e demanda estão identificar padrões de demanda, fornecer consultas adicionais para limpar atrasos e variar as modalidades de consulta (principalmente por telefone)^(25)^.

Assim como apontado pelos profissionais, a falta de recursos humanos contribui com o desequilíbrio entre a pequena oferta, a grande demanda e a fragmentação do cuidado, dificultando a oferta e garantia de um cuidado integral. A integralidade, prevista no SUS, garante que as consultas foquem no indivíduo e não se baseiem em um atendimento de queixa-conduta^(2)^. As práticas diárias da relação oferta/demanda devem, portanto, ser baseadas no paradigma da integralidade^(22)^.

A regionalização também é um fator que visa proporcionar um atendimento em saúde mais resolutivo em unidades de diferentes perfis assistenciais organizadas hierarquicamente^(26)^. Essa organização, estabelecida em uma microrregião de saúde, garante à população uma variedade de ações e serviços que não poderiam ser oferecidos de outra forma. Isso é abordado na dimensão técnica de análise do acesso^(21,24)^, que engloba o planejamento e organização de uma rede de serviços de saúde. Essa dimensão é prejudicada no contexto do CS, devido à sua distância de outros serviços de saúde e às dificuldades que a população enfrenta para conseguir acessar o local.

Segundo a dimensão política do acesso, a participação da comunidade é de extrema importância, pois, a partir da lógica da consciência sanitária, a saúde deve ser alcançada por meio de uma ação individual e coletiva, já que se trata de um direito de cidadania. Além disso, a construção do acesso deve acontecer através da participação cidadã e do controle social. O conselho local bem posicionado e ativo do CS influencia positivamente o acesso da unidade. A ação também deve partir da compreensão da população acerca da determinação social do processo de saúde-doença e dos fatores que definem a organização social das práticas de saúde^(22,24)^. Durante as reuniões de equipe, foi destacada a importância da participação do conselho local na implantação do AA, visto que os membros se posicionaram acerca de pontos positivos e negativos, assim como mudanças necessárias para melhora do acesso da população com o novo modelo.

As contradições presentes nas falas da equipe de saúde a respeito do AA vão de acordo com a dimensão simbólica do acesso, em que os sujeitos sociais, coletivamente determinados por suas representações, são formados por sujeitos históricos, que inicialmente são sujeitos coletivos^(21)^. Cada sujeito, em sua dimensão coletiva, possui uma realidade material e espiritual que se relaciona à produção de ideias, representações e consciência^(24,26)^. Nos aspectos dessa dimensão, destaca-se também a importância de a equipe se apropriar a respeito do AA e de seu referencial teórico, visto que a falta de conhecimento pode gerar dúvidas e influenciar a implantação e funcionamento do modelo^(6)^. Na implantação do modelo em unidades do Canadá, foram utilizadas como estratégias *folders*, cursos práticos e simulações, além de discussões em grupo e disponibilização de livros e referenciais^(25)^. Algumas falas e questões levantadas nas entrevistas poderiam ser resolvidas com um aprofundamento teórico. Estudo realizado durante a implantação do AA em uma UBS do município de São Paulo destacou a falta de conhecimento da equipe sobre o modelo como um dificultador de sua implantação, de modo que os profissionais entrevistados relataram dificuldade em implementar algumas inovações propostas pela equipe por falta de compreensão do modelo^(27)^.

### A enfermagem como potência para o modelo de Acesso Avançado

O papel da equipe de enfermagem na APS é amplo e diversificado, contemplando ações de prática clínica, educativa e gerencial na unidade^(2)^. O trabalho em equipe, resolubilidade da assistência, vínculo com o usuário e afinidade com o trabalho são pontos que contribuem com o acesso universal à saúde, um dos objetivos da ESF. Porém, o aumento das cargas de trabalho da equipe de enfermagem é um desafio à eficácia desse acesso, somado à sobrecarga de trabalho, excesso de demanda, problemas na estrutura física das unidades e falhas na rede de atenção^(28)^. O processo de trabalho da enfermagem, visando essa universalidade do acesso e também ao cuidado centrado nas necessidades de saúde da população, precisa ser voltado aos usuários, não aos procedimentos individuais^(28)^.

A implementação do AA tem permitido, aos usuários, o acesso à agenda multiprofissional, o que valoriza e reafirma o papel desse profissional na APS, bem como o rompimento com o modelo biomédico. Estudo realizado em 2020 em uma UBS do município de Diadema, São Paulo, destaca a autonomia dos enfermeiros como ponto essencial para a eficácia do AA na unidade. Nesse caso, a agenda dos médicos estava superlotada, e alguns casos foram direcionados à agenda dos enfermeiros, o que aumentou a resolutividade de casos e melhorou a fila de espera por atendimentos^(7)^.

A literatura tem mostrado a potência da enfermagem para o desenvolvimento do AA, com aumento da autonomia, resolutividade e envolvimento desses profissionais com as necessidades dos usuários, melhorando o acesso à APS. Estudo que avaliou a resolutividade das consultas de enfermagem com o AA evidenciou que o enfermeiro utiliza o conhecimento técnico-científico para atender às diferentes necessidades da população, sendo resolutivo em 87,7% das consultas, atingindo um percentual de resolutividade maior que o recomendado pelo Ministério da Saúde (85%)^(29)^. No cenário observado, o enfermeiro da unidade não possuía uma agenda favorável à resolutividade de demandas, e a mudança desse fator foi apontada pela equipe como essencial para a mudança para o AA.

### Limitações do estudo

A tensão entre a equipe devido à sobrecarga e divergência de opiniões pode ter afetado os dados coletados, e o início da pandemia de COVID-19 interrompeu a ida da pesquisadora ao local para coleta de dados.

### Contribuições para a área da enfermagem, saúde ou políticas públicas

Os resultados deste estudo podem ajudar na compreensão do modelo de forma prática, ressaltando seus desafios. Além disso, também contribui para melhor compreensão do potencial de ação da enfermagem dentro do contexto de acesso aos serviços de saúde e cuidado, bem como da organização do serviço e fluxo de trabalho.

## CONSIDERAÇÕES FINAIS

De acordo com a literatura existente, o modelo de AA enfrenta muitos desafios no contexto do CS estudado. Apesar de adequado às características da população da unidade, o desequilíbrio entre oferta e demanda se torna obstáculo para um cuidado adequado e realizado de forma integral.

Existe uma tensão na equipe ocasionada pela desproporção entre o tamanho da equipe e a quantidade de usuários e atendimentos, resultando em uma sobrecarga significativa. Além disso, a equipe diverge bastante entre si quanto à opinião acerca do modelo, com pontos de vista opostos acerca do modelo.

Os pacientes gostam de ter seu problema resolvido no mesmo dia, mas nem todos conseguem uma consulta devido às poucas vagas. A preocupação com a continuidade do cuidado e da assistência a pacientes crônicos também foi um ponto importante de debate, assim como o receio de que o serviço se assemelhe a um PA.

A enfermagem aparece como possível potencializadora do modelo, quando apropriada de sua capacidade de resolutividade e conhecimento técnico-científico que satisfaça as necessidades dos usuários.

O modelo de AA se destaca como uma possibilidade de organização das agendas, porém sua implantação e seu sucesso devem considerar a história e o contexto da APS em cada unidade e na política municipal, que precisam estar alinhadas aos princípios do SUS e da Saúde da Família como organizadora do modelo.

## Data Availability

Os dados de pesquisa só estão disponíveis mediante solicitação.
